# Trends of maternal health service coverage in the Democratic Republic of the Congo: a pooled cross-sectional study of MICS 2010 to 2018

**DOI:** 10.1186/s12884-021-04220-7

**Published:** 2021-11-05

**Authors:** Fuyu Guo, Xinran Qi, Huayi Xiong, Qiwei He, Tingkai Zhang, Siyu Zou, Hanyu Wang, Rie Takesue, Kun Tang

**Affiliations:** 1 Vanke School of Public Health, Tsinghua University, No. 30 Shuangqing Road, 100084 Beijing, China; 2 Health Section Programme Division, UNICEF Headquarters, 3 United Nations Plaza, New York, NY 10017 USA

**Keywords:** Antenatal care, Skilled birth attendance, Violent conflicts, The Democratic Republic of the Congo

## Abstract

**Background:**

Maternal health services are essential for reducing maternal and newborn mortality. However, maternal health service status in the Democratic Republic of the Congo (DRC) remains poorly understood. This study aims to explore the trends of antenatal care (ANC) and skilled birth attendance coverage in the past decade in the DRC.

**Methods:**

The 13,361 participants were from two rounds of Multiple Indicators Cluster Survey (MICS) conducted by the National Institute of Statistics of the Ministry of Planning of the DRC, in collaboration with the United Nations Children’s Fund (UNICEF), in 2010 and 2017-2018. A regression-based method was adopted to calculate adjusted coverage of ANC and skilled birth attendance. Subgroup analysis based on different socioeconomic status (SES) was conducted to explore the impact of domestic conflicts.

**Results:**

From 2010 to 2018, the overall weighted ANC coverage in the DRC declined from 87.3 % (95 % CI 86.1–88.0 %) to 82.4 % (95 % CI 81.1–84.0 %), while the overall weighted skilled birth attendance coverage increased from 74.2 % (95 % CI 72.5–76.0 %) to 85.2 % (95 % CI 84.1–86.0 %). Adjusted ANC coverage and adjusted skilled birth attendant coverage both declined in Kasai Oriental, but increased in Nord Kivu and Sud Kivu. In Kasai Occidental, ANC coverage declined, but skilled birth coverage increased. In the Kasai region, the largest decline in adjusted coverage of ANC was found among the poorest women. However, in the Kivu region, both the adjusted coverage of ANC and skilled birth attendance increased among the poorest women.

**Conclusions:**

Due to ongoing conflicts, there has been a systemic deterioration of maternal healthcare coverage in some regions of the DRC, particularly among people with low SES. However, in other regions, maternal healthcare services were not severely disrupted possibly due to substantial international health assistance.

**Supplementary Information:**

The online version contains supplementary material available at 10.1186/s12884-021-04220-7.

## Background

Timely and adequate maternal health services, including antenatal care (ANC), delivery care, and postnatal care (PNC), are critical to mothers and newborns [1]. Maternal health services including maternal health education, health checkups, immunization, and early identification and treatment of complications [2, 3], have been widely advocated by many international health agencies [4]. However, in low-income regions (e.g., Western and Central Africa), maternal health service coverage is far from ideal. Approximately 99 % of global preventable maternal deaths occur in low-income countries, where women lack essential childbirth services [5, 6].

The maternal mortality ratio in the Democratic Republic of the Congo (DRC) is high, estimated at 473 maternal deaths per 100,000 live births in 2017 [7], far exceeding the global target of the Sustainable Development Goal 3.1 (SDG3.1), which aims to reduce the global maternal mortality ratio to less than 70 per 100,000 live births by 2030 [8]. The United Nations Children’s Fund (UNICEF) reported that in 2018, only 82.4 % of women in the DRC have at least one antenatal care visit with skilled health care workers and only 80.1 % have skilled attendance at delivery, both below the global average level [9–11]. Although maternal health services are widely promoted in Western and Central Africa, little is known about the status and trends of maternal health service coverage in the DRC.

Since its independence in 1960, the DRC has been plagued by civil unrest and political instability and is known as one of the poorest and the most fragile countries in the world [12, 13]. There are continuing political instability and attacks in the Kivu regions, with a long-lasting conflict 2004-2012, mainly focusing on illegal exploitation of mineral resources [14]. Even after 2012, although natural resources did not cause the wars in the DRC, there was a progressive “economization” whereby the violence became increasingly motivated by profit. On the contrary, the Kasai region was relatively calm until the violence broke out in 2016 between local militants and national government forces [15]. The area became the epicenter of an intercommunal conflict that spread to neighboring provinces. At its peak, up to 1.4 million internally displaced persons, has largely subsided [16]. The United Nations reported that 5000 people died in the violent conflict, which lasted over two years [17]. The violence, frequent mass displacement, and suspension of economic and farming activities have largely affected the physiological and safety needs of people, in particular women and children [18]. Consequently, the health system in the regions has deteriorated with the disruption of essential services [19, 20].

Since 1963, UNICEF has been providing support to the DRC, including health aid on maternal and child health, to assist with the impact of the conflicts [21]. Humanitarian assistance to the DRC previously focused on the Eastern DRC, especially the Kivu region, where the conflicts were concentrated in. The Kivu region has received significant maternal and child health aid for more than a decade [22]. On the contrary, such assistance to the Kasai region was insufficient [23, 24]. Although the United Nations declared a DRC Level 3 Emergency in October 2017 in the Kasai and surrounding regions in response to the Kasai conflict, aid was not given until April 2018 [25]. Given the regional differences in the DRC, it is important to understand the trends of maternal health service coverage at regional levels under different scenarios. The present study aims to investigate the trends in the coverage of ANC and skilled birth attendance during the past decade in the DRC, with subgroup analysis by socioeconomic statuses (SES). The findings of this study will provide a picture of the trends of maternal health service and serve as a reference for future policies and programs.

## Methods

### Data Source

Data from the 2010 and 2017-2018 Multiple Indicator Cluster Survey (MICS) in the DRC was utilized. The MICS has employed a set of survey tools to generate statistically sound and internationally comparable data in 116 countries and is one of the largest international household surveys programs developed by UNICEF. To date, there have been six rounds of the MICS, for which the DRC has taken part in MICS1 (1995), MICS2 (2001), MICS4 (2010), and MICS6 (2017-2018). Among the four rounds, MICS4 and MICS6 have similar questionnaire structures. Both MICS4 and MICS6 were conducted by the National Institute of Statistics with support from UNICEF. Following a standardized protocol, multi-stage stratified sampling processes were employed to obtain samples proportional to population size in each province. Due to a governmental decentralization process started in DRC in 2015, there was an increase in the number of provinces from 11 provinces to 26 provinces, which was accounted for in the 2018 MICS. A detailed outline of corresponding provincial boundaries can be found in Additional file Table S1 [see Additional file 1]. To generate valid estimates for each province, MICS6 in 2017-2018 sampled more families. Overall, the surveys included 11,393 (MICS4) and 20,792 (MICS6) households. Further details on sampling methods can be found in the MICS4 and MICS6 summary papers [11, 26].

### Participants

**Fig. 1:**
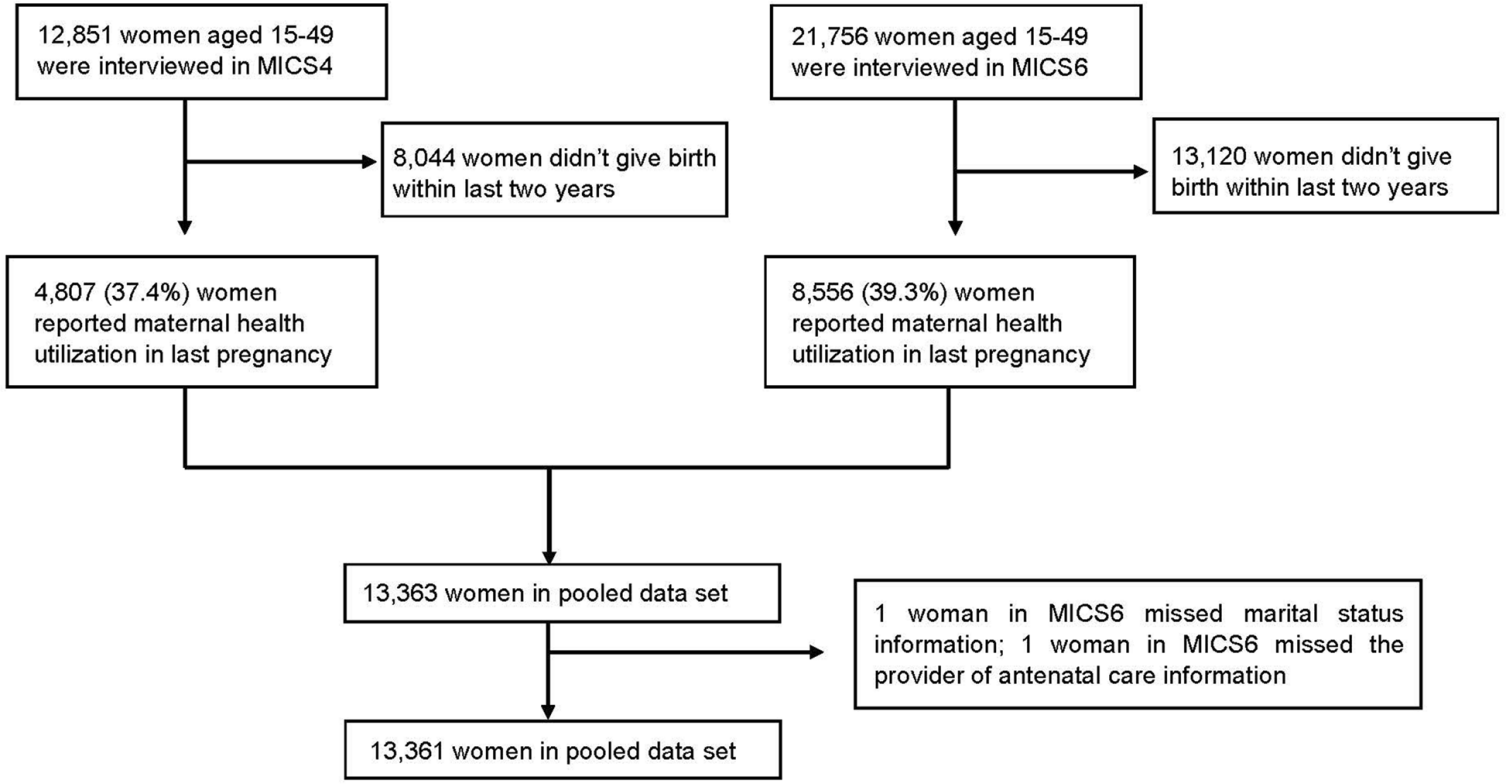
Flow diagram of the inclusion of participants

Of 12,851 and 21,756 women aged 15-49 interviewed during the MICS4 and the MICS6 surveys, only participants who had live births within the two years preceding the surveys were included to assess the coverage of ANC and skilled birth attendance in their last delivery. For complete case analysis, two women in MICS6 with missing values were excluded. A total of 13,361 women, 4,807 (36.0 %) from MICS4 and 8,554 (64.0 %) from MICS6 were included in the final analyses. The flow diagram of the inclusion or exclusion of participants is presented in Fig. 1.

### Exposures

The present study aims to explore the trends in coverage of ANC and skilled birth attendance among the provinces in the DRC. To make the province categories between MICS4 and MICS6 comparable, we recategorized the provinces in MICS6 into 11 old province categories in MICS4, including Katanga, Kasai Oriental, Kasai Occidental, Kinshasa, Bas Congo, Bandundu, Equateur, Province Orientale, Maniema, Nord Kivu, and Sud Kivu.

Additionally, we aimed to study the difference in trends of ANC coverage and skilled birth attendance among different wealth index groups. In the MICS datasets, the household wealth index was categorized into five quantiles (poorest, poorer, middle, wealthier, and wealthiest) based on an asset-based wealth index [27]. For the present study, household wealth index groups were used as proxies of participants’ SES.

### Outcomes

To ensure comparability with previous studies, we adopted the definitions from WHO [28]. Antenatal care coverage was defined as the percentage of women who utilized antenatal care provided by skilled health personnel for reasons related to pregnancy at least once during pregnancy among all women who gave birth to a live child in a given period. Qualified health personnel include physicians, nurses, and midwives. Skilled birth attendance coverage was defined as the proportion of births attended by skilled health personnel among all births. Information about whether the participants having at least one antenatal care provided by skilled birth attendants or having skilled attendance at delivery was retrieved from the MICS datasets. Data on whether antenatal care was received at least four times (ANC4) was also available. However, as the service providers of each visit were not clearly specified and there was a large number of missing data on visit times, the prevalence of ANC4 is presented in Additional file 2 as a description but is not included in the main analysis.

### Covariates

Based on reviewed literature [29–33], we chose women’s age, educational attainment, marital status, household heads’ sex, and residence region as covariates. Women were classified into four age groups:15-19, 20-29, 30-39, and 40-49. Women’s educational attainment was categorized into three groups: below the primary school, primary school, and secondary or higher school. Women’s marital status information was also retrieved from the original datasets and re-categorized into two groups: currently single and married or living with a partner. For the residence region, it is worth noting that in MICS4 and MICS6, no participants were categorized as living in rural regions in Kinshasa.

### Statistical analysis

Descriptive statistics were calculated to show the characteristics of survey participants. The overall coverage of ANC and skilled birth attendance was calculated using sampling weights provided in the MICS datasets. The weighted coverage was then compared with survey findings in the UNICEF reports [11, 26] to validate our analyses. A logistic regression-based adjusted prevalence method was used to calculate adjusted coverage of ANC and skilled birth attendance. Details for the method were described elsewhere [34, 35]. Briefly, the probability of the event (binary variable) for each individual was firstly modeled by constructing a logistic regression in which independent variables were the group variable such as province and the chosen adjusted variables (e.g., educational attainment, wealth index). Secondly, for each participant in the datasets, the probability as well as the standard error of the event was predicted using the model. Lastly, for each group, the mean of the probability of the event among the participants within the group was calculated. In this study, the coverage of ANC and skilled birth attendance for participants from different provinces in 2010 and 2018 were calculated, adjusted for women’s age, educational attainment, marital status, household heads’ sex, residence region, and household wealth status. Maps were drawn to visualize the trends in coverage of adjusted ANC and adjusted skilled birth attendance from 2010 to 2018. 

To further compare the trends between regions with conflict but who were largely unassisted (low humanitarian assistance, especially for maternal and child health, i.e., the Kasai region), regions with conflict but who were assisted (i.e., the Kivu region), and regions without such extensive conflict during 2010 and 2018 (other provinces), subgroup analyses were applied. In each region, adjusted ANC coverage and adjusted skilled birth attendance coverage with 95 % confidence intervals in different wealth index groups were calculated. We chose the other provinces as a control group to reflect the influence of large conflict in the Kasai region and the Kivu region, as well as the influence of international health aid. Provinces around the Kasai region and the Kivu region might also be influenced by the conflict. To check if any province would significantly impact the results of the control group, we did a sensitivity analysis by dropping a province out of the control group in turns (Additional file 3).No significantly different trends between 2010 and 2018 were found in this analysis. Thus, to include as many as samples as possible as controls, we finally decided to aggregate the other provinces in a control group. Data were processed and analyzed using R 4.0.0 (R Core Team, 2020).

## Results

### Participants’ demographic and socioeconomic characteristics

A total of 13,133 women were included in the analyses, with 4,807 from MICS4 (2010) and 8,554 from MICS6 (2017-2018). Of all participants, roughly half were aged 20-29 years, with 54.8 % participants in 2010 and 45.1 % in 2018. Only 37.5 % of women had attended secondary school or higher. For women’s marital status, about 87.0 % of them were married or currently living with a partner, with no significant difference between 2010 and 2018. Most women came from the rural regions in the DRC, with 64.9 % in 2010 and 73.5 % in 2018. In 2010, the distribution of the SES of women was roughly even among five categories from the poorest to the wealthiest households. However, in 2018 more women were considered to live in the poorest and poorer households. Details for participants’ demographic and socioeconomic characteristics are shown in Table 1.

**Table 1:** Characteristics of the study participants by survey year

Variables	2010 (N=4,807)			2018 (N=8,554)		
	**Numbers**	**% (unweighted)**^**a**^	**% (weighted)** ^**b**^	**Numbers**	**% (unweighted)**^**a**^	**% (weighted)** ^**b**^
**Antenatal care coverage**	4,159	86.5	87.3	6,554	76.6	82.4
**Skilled birth attendance coverage**	3,686	76.7	74.2	6,361	74.4	85.2
**Age**						
15-19	450	9.4	9.8	873	10.2	10.1
20-29	2,632	54.8	54.7	3,858	45.1	45.0
30-39	1,401	29.1	28.9	3,197	37.4	37.5
40-49	324	6.7	6.7	626	7.3	7.4
**Educational attainment**						
Below primary	1,066	22.2	23.4	1,872	21.9	17.6
Primary	2,008	41.8	44.2	3,408	39.8	34.4
Secondary or higher	1,733	36.1	32.4	3,274	38.3	47.8
**Marital status**						
Currently Single	622	13.1	13.3	1,108	13.0	15.1
Married or living with a partner	4,137	86.9	86.7	7,446	87.0	84.9
**Residential region**						
Rural	3,100	64.9	74.6	6,287	73.5	61.6
Urban	1,707	35.1	25.3	2,267	26.5	38.4
**Wealth index group**						
Poorest	951	19.5	22.3	2,873	33.6	24.3
Poorer	962	20.0	21.9	2,270	26.5	21.6
Middle	939	19.7	20.3	1,713	20.0	19.7
Wealthier	1,069	22.2	19.7	1,160	13.6	19.4
Wealthiest	886	18.4	15.8	538	6.3	14.9
**Province**						
Bandundu	417	8.7	11.0	840	9.8	12.0
Bas Congo	346	7.2	6.0	263	3.1	6.2
Equateur	477	9.9	12.1	1,665	19.5	6.5
Kasai Occidental	453	9.4	6.3	759	8.9	8.7
Kasai Oriental	462	9.6	8.2	1,129	13.2	8.6
Katanga	552	11.5	18.3	1,422	16.6	17.0
Kinshasa	372	7.7	9.1	255	3.0	10.2
Nord Kivu	453	9.4	8.7	339	4.0	8.3
Sud Kivu	484	10.1	8.2	389	4.5	8.9
Maniema	424	8.8	2.9	324	3.8	1.4
Province Orientale	367	7.6	9.2	1,169	13.7	12.2
**Household head’s sex**						
Male	4,193	87.2	87.5	6,514	76.2	75.9
Female	614	12.8	12.5	2,040	23.8	24.1

**Notes**:

^a^ Unweighted proportion is the fraction of the number of the group over the whole population in this study

^b^ Weighted proportion was based on the unweighted proportion and further weighted by sampling weights provided in the MICS dataset

### The trend of prevalence of maternal health care services from 2010 to 2018

The overall weighted ANC coverage decreased from 87.3 % (95 % CI 86.1–88.0 %) to 82.4 % (95 % CI 81.1–84.0 %) in 2018, and the weighted skilled birth attendance coverage increased from 74.2 % (95 % CI 72.5–76.0 %) to 85.2 % (95 % CI 84.1–86.0 %) in 2018 [Table 1].

**Fig. 2:**
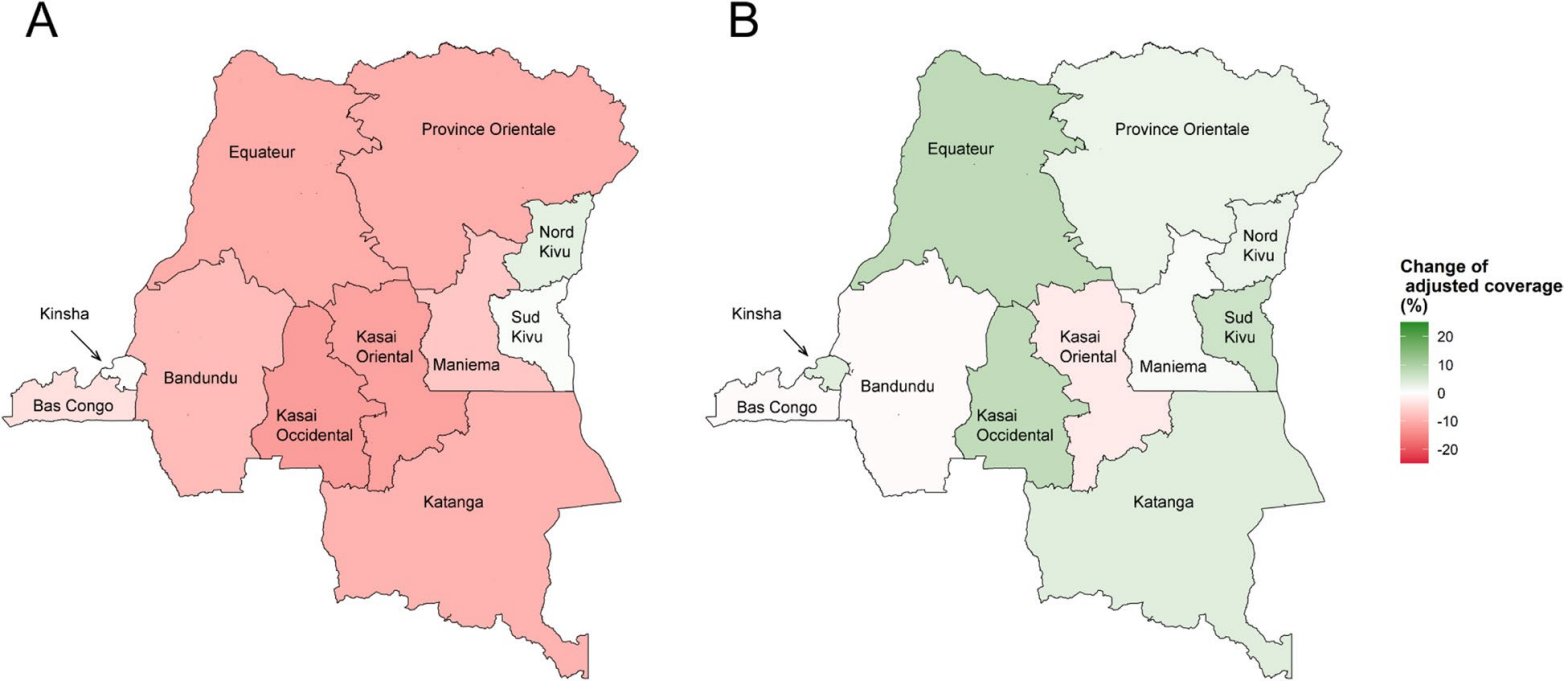
The change of adjusted coverage of maternal health services in the DRC 2010 – 2018. Legends: **a** Panel A represents the change of adjusted coverage of antenatal care and Panel B represents the change of adjusted coverage of skilled birth attendance. **b** Positive figures in the legend which was shown as green on the map present the increase of adjusted coverage from 2010 to 2018. Negative figures (Red on the map) present the decrease of the adjusted coverage. **c** The adjusted coverage was calculated after adjusting for women’s age, educational attainment, marital status, household heads’ sex, residential region, and household wealth index groups

As shown in Fig. 2*(A)*, the adjusted ANC coverage declined in most provinces in the DRC, except in Kinshasa, Nord Kivu, and Sud Kivu. In Nord Kivu, the adjusted ANC coverage was 95.1 % (95 % CI 92.8–97.5 %) in 2010, and 98.2 % (95 % CI 96.7–99.8 %) in 2018. In Sud Kivu, the adjusted ANC coverage was 89.5 % (95 % CI 86.1–92.8 %) in 2010 and 90.0 % (95 % CI 86.4–93.5 %) in 2018, as shown in Table 2. The trends of adjusted ANC coverage were consistent between rural and urban regions in the DRC

**Table 2:** Adjusted coverages of maternal health service in the DRC 2010 - 2018^a^

Province	Antenatal Care				Skilled Birth Attendance			
	2010 (N=4,807)		2018 (N=8,554)		2010 (N=4,807)		2018 (N=8,554)	
	Percentage (%)	95 % CI (%)	Percentage (%)	95 % CI (%)	Percentage (%)	95 % CI (%)	Percentage (%)	95 % CI (%)
Bandundu	90.4	87.1-93.7	82.0	78.5-85.5	84.7	80.4-88.9	84.0	80.8-87.3
Bas Congo	95.4	92.8-98.0	91.6	87.8-95.4	94.5	91.3-97.7	93.9	90.5-97.3
Equateur	84.3	80.3-88.2	74.1	70.5-77.7	50.9	45.6-56.2	58.6	54.5-62.8
Kasai Occidental	81.0	76.7-85.4	68.8	64.2-73.3	69.5	64.4-74.7	77.1	73.1-81.0
Kasai Oriental	79.4	74.9-84.0	67.9	63.7-72.2	69.5	64.3-74.7	66.9	62.8-71.0
Katanga	80.1	75.6-84.5	70.7	66.7-74.8	63.0	57.9-68.2	66.7	62.7-70.6
Kinshasa	94.6	92.1-97.1	94.9	92.0-97.8	96.0	93.4-98.6	99.6	98.6-100.0
Maniema	76.7	71.7-81.6	69.8	63.9-75.6	70.5	65.2-75.8	71.3	65.6-77.0
Nord Kivu	95.1	92.8-97.5	98.2	96.7-99.8	95.4	93.0-97.7	97.6	95.8-99.5
Province Orientale	90.7	87.1-94.4	80.8	77.3-84.2	82.6	77.6-87.5	84.9	82.0-87.9
Sud Kivu	89.5	86.1-92.8	90.0	86.4-93.5	80.2	75.6-84.7	86.1	81.9-90.3

Notes:

a Adjusted prevalence was calculated after adjusting for women’s age, educational attainment, marital status, household heads’ sex, residential region, and household wealth index group

Figure 2*(B)* shows the trend of adjusted skilled birth attendance coverage in the DRC from 2010 to 2018. In contrast to the adjusted ANC coverage, the adjusted skilled birth attendance coverage increased in most provinces. However, there was still a decrease in Kasai Oriental, with the adjusted skilled birth attendance coverage decreasing from 69.5 % (95 % CI 64.3–74.7 %) in 2010 to 66.9 % (95 % CI 62.8–71.0 %) in 2018 (Table 2).

### The prevalence of maternal health care service for women in different SES

**Fig. 3:**
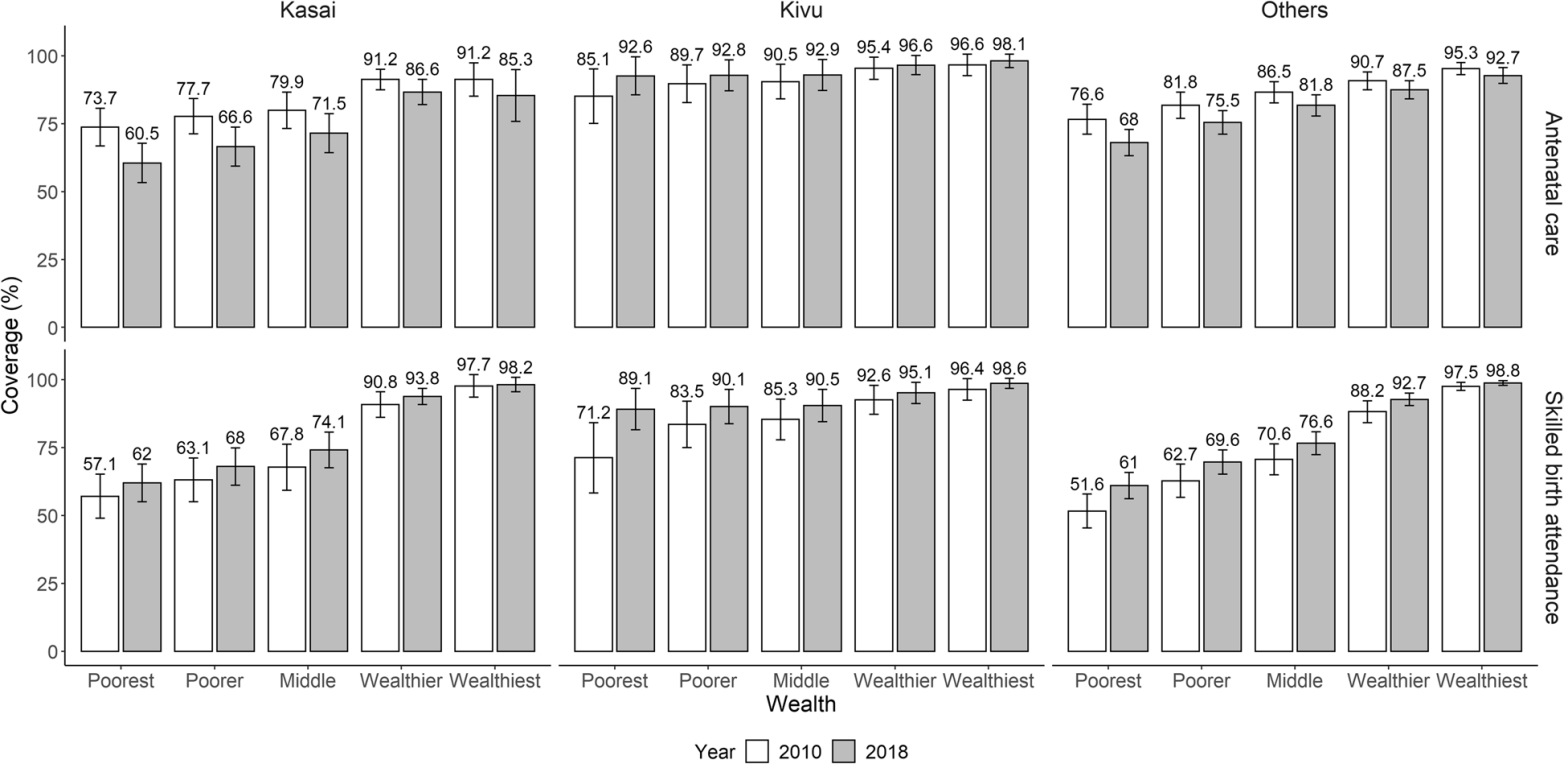
The trends of adjusted coverage of maternal health service among different SES 2010 – 2018. Legends: The adjusted coverage was calculated after adjusting for women’s age, educational attainment, marital status, household heads’ sex, residential region, and household wealth index groups

Figure 3 presents the adjusted ANC coverage and adjusted skilled birth attendance coverage for women from different wealth index groups in the Kasai region, the Kivu region, and the other provinces in the DRC. For adjusted ANC coverage, a general decreasing trend from 2010 to 2018 was observed in the Kasai region and other provinces in the DRC. The most significant decline of adjusted ANC coverage in the Kasai region was in the poorest women from 73.7 % (95 % CI 66.8–80.6 %) in 2010 to 60.5 % (95 % CI 53.3–67.7 %) in 2018. In contrast to most provinces in the DRC, there was an increasing trend of adjusted ANC coverage for all groups in the Kivu region. Within each wealth index group in 2018, the adjusted ANC coverage in the Kivu region was higher than in the other provinces. When it comes to the adjusted skilled birth attendance coverage, there was a general increasing trend in the DRC. The largest increment was observed women in the Kivu region from 71.2 % (95 % CI 58.2–84.2 %) in 2010 to 89.1 % (95 % CI 81.5–96.8 %) in 2018. Besides, except for the wealthiest women, the adjusted skilled birth attendance coverage in the Kivu region was higher than in the other provinces for all wealth index groups in 2018.

## Discussion

In the present study, we observed a decline of ANC coverage in the DRC from 2010 to 2018, while we observed an increase in skilled birth attendance during the same time period. Both ANC coverage and skilled birth attendance coverage were found to have declined in Kasai Oriental, while both were found to have increased in the Kivu region. The largest decline in ANC was observed among women in the Kasai region. However, for women in the Kivu region, those in the lowest SES group saw a significant increase in both ANC coverage and skilled birth attendance coverage.

The DRC, with the most severe health challenge of its high maternal mortality ratio, has received a large amount of health assistance from various international organizations, of which a significant share was devoted to maternal and reproductive health interventions [36–38]. However, given the political instability and armed conflict, not all health assistance programs were effective. The results of this study suggested that skilled birth attendance coverage has increased from 2010 to 2018, which was consistent with the overall trend in Western and Central Africa [10]. This increase illustrated great progress made by the programs targeting improving the delivery situation [39, 40]. However, the ANC coverage was found to decrease in most regions, with the country-level coverage below the world average[9]. The conflicts in the country and the lack of funding for the maternal health service from the DRC government contributed to the decline. ANC plays a crucial role in promoting maternal health [41–43]. The low level of ANC coverage suggests that more efforts and targeted public health interventions are needed to promote maternal and child health in the DRC.

It was found that the ANC coverage and skilled birth attendance coverage both decreased in Kasai Oriental, indicating a systemic problem in the province. Due to the horrific violence that erupted in 2016, women in the Kasai region were reported to have limited access to essential health care [23]. Previous studies found that serious atrocities in the Kasai region forced people to leave their homes and even their country [44]. While some of them are returning, the situation in the Kasai remains volatile as the wider political situation continues to deteriorate. Maternal health service coverage there was inadequate. Unlike the Kasai region, where health intervention programs have not been fully implemented yet, the Kivu region has received continuous international health assistance for sexual and reproductive health. A previous study showed that the operational capacity score for primary emergency obstetric care in Nord Kivu and Sud Kivu ranked second and third in the whole country, respectively, making them just behind Kinshasa, the capital province of the DRC [45]. The Kivu region also had the highest percentage of health facilities with family planning services available [38]. Furthermore, the Kivu region consists of bigger cities with higher population density compared to other provinces, potentially providing residents with greater access to healthcare facilities [46]. Consistent with previous findings, the present study found that the coverage of ANC and skilled birth attendance increased from 2010 to 2018 in the Kivu region, given the circumstance of an overall decline of ANC coverage at the country level. Despite the long-lasting instabilities, which may have caused difficulties for women to have access to adequate maternal health care in the region, such results demonstrated the potential efficiency of health aid in the Kivu region.

Additionally, health interventions and social changes usually have differentiated impacts concerning health service coverage based on people’s SES [47]. It was reported that the domestic conflict in the Kasai region had affected 170 health centers as of October 2017, leaving women with limited access to health care [24]. Without adequate local health care services, many women have to travel to distant health centers to seek maternal health services. Hence, traveling costs have become an obstacle for low-income families. On the other hand, although influenced by longer conflicts durations, the situation of women with low SES in the Kivu region has improved over the years. The coverage of skilled birth attendance in women with the lowest SES was found to increase by one quarter, more extensive than most of their wealthier peers. Although disparities in maternal health care coverage among different SES groups still exist, it is gradually being reduced in the Kivu region. The differing trends between the Kasai and Kivu regions demonstrate the critical role of international health aid. Health centers with better access to international health aid may be able to provide health services with better accessibility and affordability to vulnerable populations such as mothers and children.

The results of this study also indicate that ANC and skilled birth attendance coverage in the Kivu region was higher compared to other provinces with little or no conflicts. Although it demonstrates the effectiveness of the health interventions, another question arises -- whether further health resources should be reallocated to other regions of the country? On the one hand, Kivu is still in conflict and is facing the consequential impacts of an Ebola outbreak from February to May 2021 [48]. Continued health aid is needed to keep the high level of maternal health service coverage in the area. On the other hand, maternal health coverage in other provinces in the DRC was much lower than that in the Kivu region. Accessible and affordable health service was also in urgent need for those regions. The vertical health aid programs targeting specific conflict areas may largely ignore the poor situation in other parts of the country, and a horizontal investment of the country’s overall health system should be considered as a priority.

### Strength and limitations

To our knowledge, this article is one of the first studies exploring the temporal trends of maternal health service coverage in the DRC by using two rounds of nationally representative surveys of the MICS4 and MICS6. We applied adjusted prevalence to provide an unbiased estimate of the health service coverage in DRC by region and individual’s socioeconomic status. Besides, our results illustrate how conflicts impact women of different SES in the Kasai and Kivu regions and the potential role of international health aid in conflicting areas.

There are several limitations to this study. Firstly, without comparable indicators in MICS1 and MICS2 in the DRC, long-term trends of maternal health service coverage could not be entirely assessed in this study. Secondly, due to the re-division of the provinces in 2015, the sampling processes were slightly different between MICS4 and MICS6 at the province level. To make it comparable between the two surveys, we re-categorized the provinces in MICS6 into those in MICS4. The low spatial resolution caused by combining current provinces together makes our findings less precise in large provinces such as Equateur province which was divided into 4 smaller provinces in 2015. Thirdly, other confounders such as the supply of skilled birth attendants, the number of health facilities in each province, and health expenditure are not fully measured in the MICS survey, and not included in this study. Lastly, the method of adjusted prevalence has its limitations. Although it can compare groups efficiently and achieve the goals of unbiased estimation of population prevalence, it is substantially more challenging to attain when the sampling strategy is more complicated than the simple random sampling schemes. Also, the variation of estimates for groups with small sizes is relatively large.

## Conclusions

Based on the analysis of two rounds of MICS data in the DRC, opposite trends in the coverages of ANC and skilled birth attendance were found between 2010 and 2018. Overall, while the skilled birth attendance coverage increased, the ANC coverage decreased over the years. In Kasai-Oriental, where conflicts newly erupted, the situation became worse for both services. Women with lower SES were found to have significantly less access to maternal health services. Although influenced by long-lasting conflicts, the Kivu region saw an increase both in ANC coverage and skilled birth attendance coverage. The disparities between women in higher and lower SES have also slightly reduced from 2010 to 2018 in the Kivu region. There was a higher maternal health service coverage in the Kivu region despite conflicts, suggesting a successful implementation of an international health aid program. However, this raised further questions of the effective resource allocation and the role of international health aid in supporting vertical programs versus horizontal programs.

## Supplementary Information

**Additional file 1.** Approximate corresponding boundaries of provinces between MICS 2010 and MICS 2017-2018. On 9 January 2015, the National Assembly of the DRC passed a law on the new administrative divisions of the country. To make the province variables in MICS6 (2017-2018) comparable with the data in MICS4 (2010), we re-categorized the provinces in MICS6 into the division system in 2010. Table 1 presents the correspondence of provinces between 2010 and 2018.

**Additional file 2.** Weighted coverage in the country-level and adjusted coverage in the province-level of receiving at least four times antenatal care in the DRC in 2010 and 2018.

**Additional file 3.** Sensitivity analysis of the adjusted coverage in the other provinces. Each province excpet those in the Kasai reigon and the Kivu region was dropped out in turns to check if any specific province impacted the adjusted coverage of the maternal health service in the other provinces significantly. These provinces includes: Kinshasa, Base Congo, Bandundu, Equateur, Orientale, Maniema, and Katanga.

**Additional file 4.** Adjusted coverage of maternal health service in urban and rural regions of DRC from 2010 to 2018. Detailed adjusted coverage of maternal health services in urban and rural regions of the DRC from 2010 to 2018.

## Data Availability

The datasets generated and/or analyzed during the current study are available in the UNICEF MICS survey home page, [https://mics.unicef.org/surveys/].

## Abbreviations

DRC: the Democratic Republic of the Congo
ANC: antenatal care
UNICEF: United Nations Children’s Fund
MICS: Multiple Indicators Cluster Survey
SES: socioeconomic status
