# Supplementary material for: Trends of maternal health service coverage in the Democratic Republic of the Congo: a pooled cross-sectional study of MICS 2010 to 2018
Source: BMC Pregnancy Childbirth. 2021 Nov 5;21:748. doi: 10.1186/s12884-021-04220-7 (PMC8569966; doi:10.1186/s12884-021-04220-7)
Supplement: Supplementary file 1 — Additional file 1. Approximate corresponding boundaries of provinces between MICS 2010 and MICS 2017-2018. On 9 January 2015, the National Assembly of the DRC passed a law on the new administrative divisions of the country. To make the province variables in MICS6 (2017-2018) comparable with the data in MICS4 (2010), we re-categorized the provinces in MICS6 into the division system in 2010. Table 1 presents the correspondence of provinces between 2010 and 2018. [file 12884_2021_4220_MOESM1_ESM.docx]

On 9 January 2015, the National Assembly of the DRC passed a law on the new administrative divisions of the country. To make the province variables in MICS6 (2017-2018) comparable with the data in MICS4 (2010), we re-categorized the provinces in MICS6 into the division system in 2010. Table 1 presents the correspondence of provinces between 2010 and 2018.

**Table S1** Approximate correspondence of provinces between MICS 2010 and MICS 2017-2018

| Provinces in MICS4 (2010) | Provinces in MICS6 (2017-2018) |
| --- | --- |
| Katanga | Tanganyika |
|  | Haut Lomami |
|  | Lualaba |
|  | Haut Katanga |
| Kasai Oriental | Lomami |
|  | Sankuru |
|  | Kasai Oriental |
| Kasai Occidental | Kasai Central |
|  | Kasai |
| Kinshasa | Kinshasa |
| Bas Congo | Kongo Central |
| Bandundu | Kwango |
|  | Kwilu |
|  | Mai Ndombe |
| Equateur | Equateur |
|  | Tshuapa |
|  | Mongala |
|  | Notd Ubangi |
|  | Sud Ubangi |
| Province Orientale | Bas-Uele |
|  | Haut-Uele |
|  | Ituri |
|  | Tshopo |
| Maniema | Maniema |
| Nord Kivu | Nord Kivu |
| Sud Kivu | Sud Kivu |
