# Supplementary material for: Trends of maternal health service coverage in the Democratic Republic of the Congo: a pooled cross-sectional study of MICS 2010 to 2018
Source: BMC Pregnancy Childbirth. 2021 Nov 5;21:748. doi: 10.1186/s12884-021-04220-7 (PMC8569966; doi:10.1186/s12884-021-04220-7)
Supplement: Supplementary file 2 — Additional file 2. Weighted coverage in the country-level and adjusted coverage in the province-level of receiving at least four times antenatal care in the DRC in 2010 and 2018. [file 12884_2021_4220_MOESM2_ESM.docx]

**Table S1** Coverage of antenatal care (at least 4 times) in the DRC (2010-2018)

| Results | Antenatal Care Visits >= 4 | | | | | |
| --- | --- | --- | --- | --- | --- | --- |
|  | 2010 (N=4,807) | | | 2018 (N=8,554) | | |
|  | **Numbers** | **% (unweighted)^a^** | **% (weighted)^b^** | **Numbers** | **% (unweighted)^a^** | **% (weighted)^b^** |
| At least 4 times | 2,095 | 43.6 | 43.8 | 3,225 | 37.7 | 43.1 |
| Less than 4 times | 2,067 | 43.0 | 43.5 | 3,390 | 39.6 | 39.9 |
| Unknown | 645 | 13.4 | 12.6 | 1,939 | 22.7 | 17.0 |

**Notes:**

a. Unweighted proportion is the fraction of the number of the group over the whole population in this study.

b. Weighted proportion was based on the unweighted proportion and further weighted by sampling weights provided in the MICS dataset.

**Table S2** Adjusted coverage of antenatal care (at least 4) in the DRC 2010 - 2018^a^

| Province | Antenatal Care | | | |
| --- | --- | --- | --- | --- |
|  | 2010 (N=4,807) | | 2018 (N=8,554) | |
|  | Percentage (%) | 95% CI (%) | Percentage (%) | 95% CI (%) |
| Bandundu | 42.0 | 36.4-47.5 | 37.3 | 32.9-41.6 |
| Bas Congo | 47.4 | 41.3-53.5 | 33.8 | 27.4-40.2 |
| Equateur | 47.4 | 42.1-52.7 | 35.3 | 31.7-39.0 |
| Kasai Occidental | 43.9 | 38.6-49.3 | 36.1 | 31.7-40.5 |
| Kasai Oriental | 45.2 | 39.8-50.6 | 32.3 | 28.5-36.2 |
| Katanga | 35.3 | 30.4-40.3 | 37.9 | 34.0-41.8 |
| Kinshasa | 71.2 | 66.1-76.4 | 69.4 | 63.2-75.6 |
| Maniema | 30.7 | 25.6-35.8 | 20.7 | 15.8-25.6 |
| Nord Kivu | 46.4 | 40.8-51.9 | 51.0 | 44.9-57.1 |
| Province Orientale | 52.0 | 46.1-58.0 | 38.5 | 34.4-42.6 |
| Sud Kivu | 27.1 | 22.3-31.8 | 48.8 | 43.0-54.7 |

Notes:

a Data in this table present the adjusted coverage unless stated. Standardized prevalence was calculated after adjusting for women’s age, educational attainment, marital status, household heads’ sex, residential region, and household wealth index group.
