# Supplementary material for: Trends of maternal health service coverage in the Democratic Republic of the Congo: a pooled cross-sectional study of MICS 2010 to 2018
Source: BMC Pregnancy Childbirth. 2021 Nov 5;21:748. doi: 10.1186/s12884-021-04220-7 (PMC8569966; doi:10.1186/s12884-021-04220-7)
Supplement: Supplementary file 3 — Additional file 3. Sensitivity analysis of the adjusted coverage in the other provinces. Each province excpet those in the Kasai reigon and the Kivu region was dropped out in turns to check if any specific province impacted the adjusted coverage of the maternal health service in the other provinces significantly. These provinces includes: Kinshasa, Base Congo, Bandundu, Equateur, Orientale, Maniema, and Katanga. [file 12884_2021_4220_MOESM3_ESM.docx]

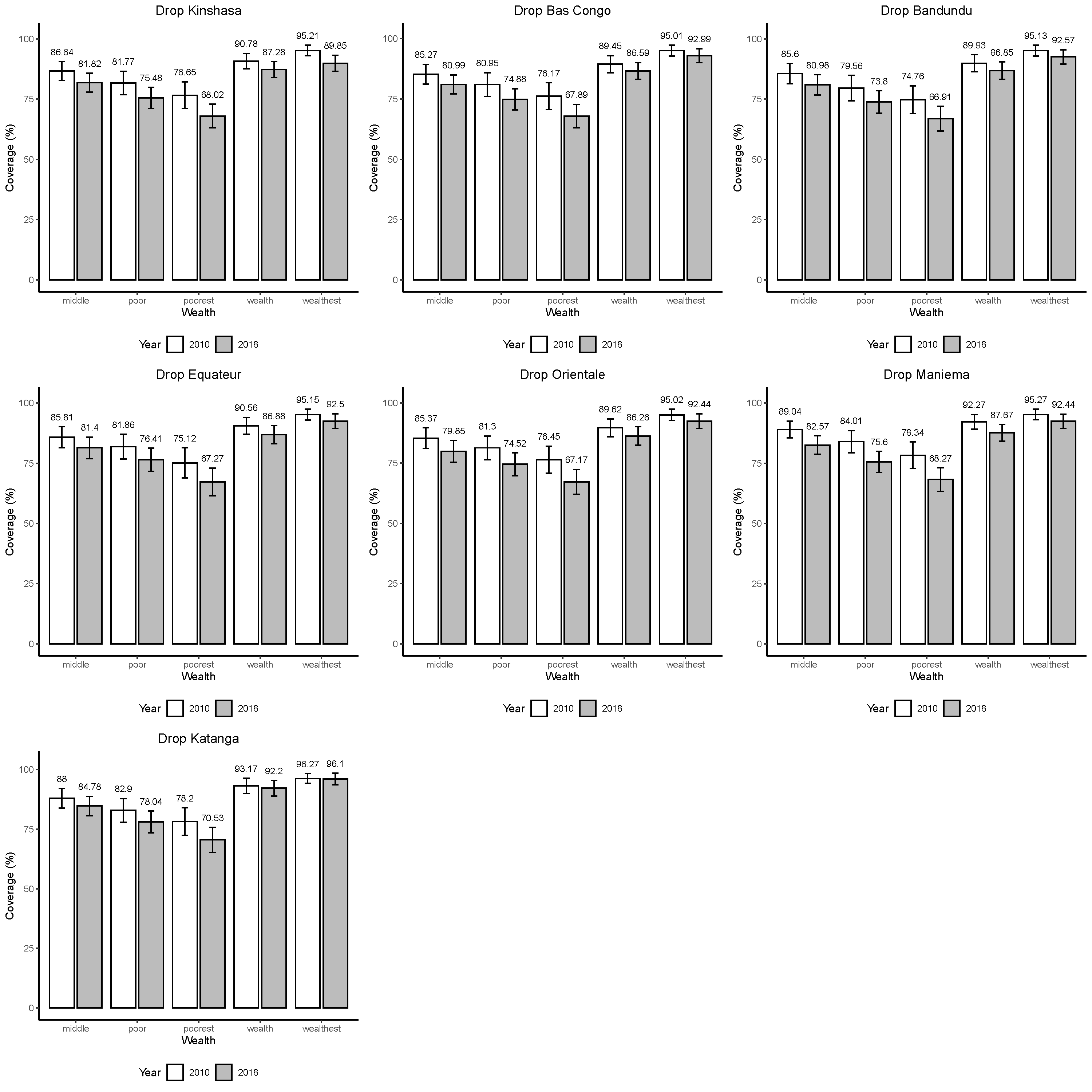
Fig S1. The trends of adjusted coverage of antenatal care among different SES 2010 – 2018 in the provinces except the Kasai region and the Kivu region.^a^

a. A province was dropped once in turns to check if any specific province impacted the results of the whole significantly.


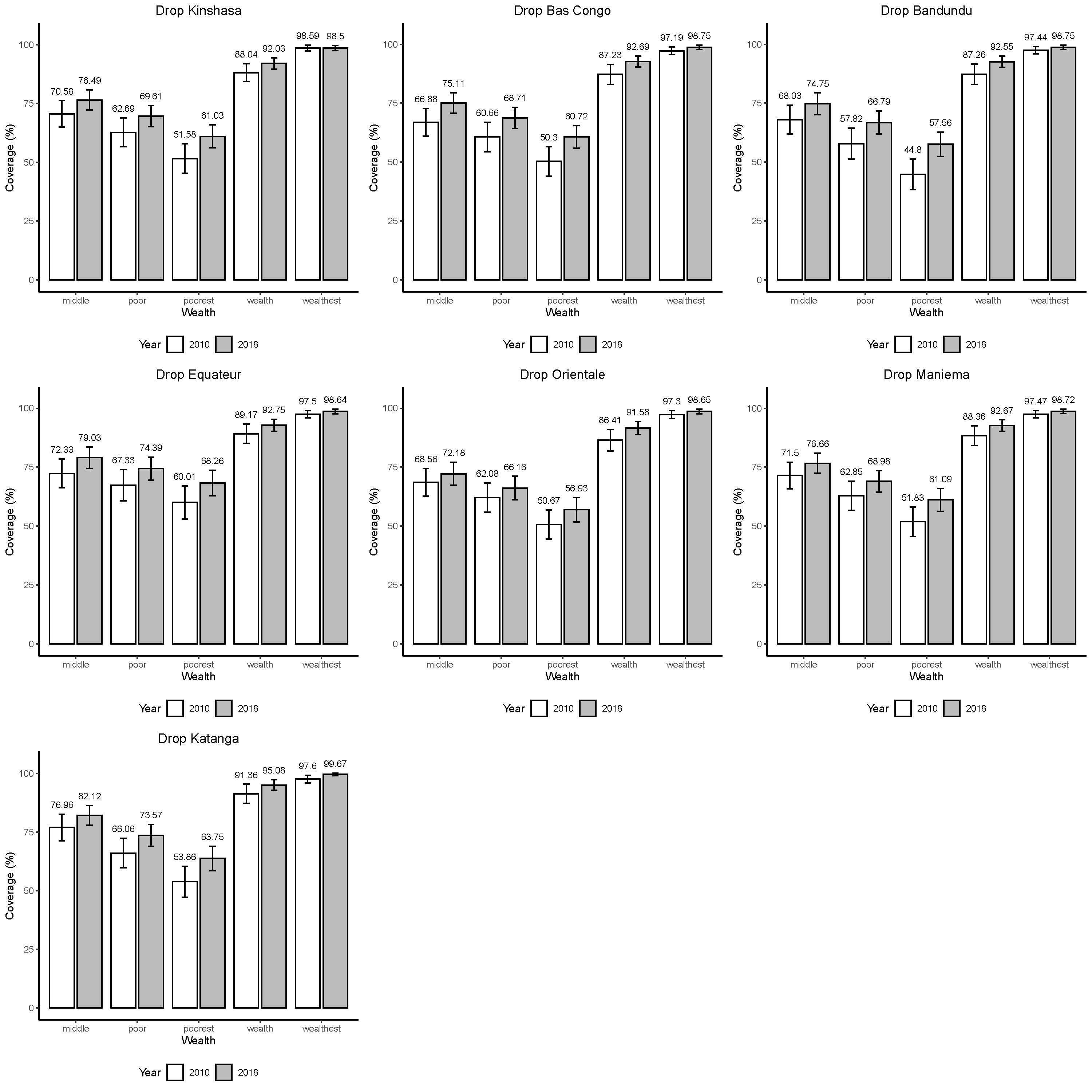
Fig S2. The trends of adjusted coverage of skilled birth attendance among different SES 2010 – 2018 in the provinces except the Kasai region and the Kivu region.^a^

a. A province was dropped once in turns to check if any specific province impacted the results of the whole significantly.
