# Supplementary material for: Trends of maternal health service coverage in the Democratic Republic of the Congo: a pooled cross-sectional study of MICS 2010 to 2018
Source: BMC Pregnancy Childbirth. 2021 Nov 5;21:748. doi: 10.1186/s12884-021-04220-7 (PMC8569966; doi:10.1186/s12884-021-04220-7)
Supplement: Supplementary file 4 — Additional file 4. Adjusted coverage of maternal health service in urban and rural regions of DRC from 2010 to 2018. Detailed adjusted coverage of maternal health services in urban and rural regions of the DRC from 2010 to 2018. [file 12884_2021_4220_MOESM4_ESM.docx]

**Table S2** Adjusted coverage of maternal health service in rural regions of DRC from 2010 to 2018^a^

| Province | Antenatal Care | | | | Skilled birth attendance | | | |
| --- | --- | --- | --- | --- | --- | --- | --- | --- |
|  | 2010 (N=4,759) | | 2018 (N=8,554) | | 2010 (N=4,759) | | 2018 (N=8,554) | |
|  | Percentage (%) | 95% CI (%) | Percentage (%) | 95% CI (%) | Percentage (%) | 95% CI (%) | Percentage (%) | 95% CI (%) |
| Bandundu | 88.3 | 84.5-92.1 | 80.7 | 77.1-84.3 | 79.8 | 74.8-84.7 | 82.1 | 78.7-85.6 |
| Bas Congo | 94.1 | 91.0-97.2 | 90.4 | 86.2-94.5 | 92.1 | 88.3-96.0 | 92.5 | 88.7-96.3 |
| Equateur | 81.7 | 77.4-86.0 | 72.1 | 68.4-75.8 | 40.8 | 35.2-46.4 | 54.1 | 49.8-58.4 |
| Kasai Occidental | 77.3 | 72.5-82.2 | 65.9 | 61.2-70.6 | 61.1 | 55.3-67.0 | 73.5 | 69.3-77.8 |
| Kasai Oriental | 75.3 | 70.2-80.4 | 60.5 | 55.9-65.1 | 60.6 | 54.7-66.6 | 54.4 | 49.7-59.2 |
| Katanga | 74.9 | 69.9-79.9 | 62.3 | 57.8-66.7 | 49.1 | 43.1-55.0 | 51.9 | 47.2-56.6 |
| Kinshasa |  |  |  |  |  |  |  |  |
| Maniema | 72.1 | 66.6-77.5 | 67.3 | 61.3-73.4 | 61.4 | 55.3-67.5 | 67.3 | 61.2-73.5 |
| Nord Kivu | 94.3 | 91.7-96.9 | 97.9 | 96.2-99.7 | 94.1 | 91.3-96.8 | 97.1 | 95.0-99.2 |
| Province Orientale | 86.7 | 82.1-91.3 | 78.3 | 74.7-81.9 | 71.2 | 64.7-77.8 | 81.5 | 78.2-84.8 |
| Sud Kivu | 87.7 | 83.9-91.4 | 88.5 | 84.7-92.4 | 74.8 | 69.6-80.0 | 82.9 | 78.2-87.6 |

Notes:

a. Data in the table present the adjusted coverage unless stated. Standardized prevalence was calculated after adjusting for women’s age, education attainment, marital status, household heads’ sex, residence region, and household wealth index group.

**Table S3** Adjusted coverage of maternal health service in the urban region of DRC from 2010 to 2018^a^

| Province | Antenatal Care | | | | Skilled birth attendance | | | |
| --- | --- | --- | --- | --- | --- | --- | --- | --- |
|  | 2010 | | 2018 | | 2010 | | 2018 | |
|  | Percentage (%) | 95% CI (%) | Percentage (%) | 95% CI (%) | Percentage (%) | 95% CI (%) | Percentage (%) | 95% CI (%) |
| Bandundu | 95.9 | 94.2-97.5 | 89.7 | 87.0-92.4 | 97.0 | 95.9-98.2 | 95.4 | 94.0-96.8 |
| Bas Congo | 98.1 | 96.9-99.2 | 96.6 | 94.8-98.3 | 99.5 | 99.1-99.8 | 99.3 | 98.8-99.8 |
| Equateur | 92.3 | 89.9-94.8 | 85.8 | 82.8-88.7 | 82.2 | 78.0-86.5 | 85.5 | 82.5-88.5 |
| Kasai Occidental | 90.5 | 87.6-93.3 | 81.9 | 78.0-85.7 | 91.1 | 88.5-93.8 | 93.2 | 91.3-95.1 |
| Kasai Oriental | 90.5 | 87.6-93.3 | 80.5 | 76.9-84.1 | 93.0 | 90.7-95.3 | 87.9 | 85.2-90.5 |
| Katanga | 90.1 | 87.1-93.0 | 85.5 | 82.4-88.6 | 90.1 | 87.0-93.2 | 92.4 | 90.4-94.4 |
| Kinshasa | 94.6 | 92.1-97.1 | 94.9 | 92.0-97.8 | 96.0 | 93.4-98.6 | 99.6 | 98.6-100.0 |
| Maniema | 87.2 | 83.7-90.7 | 81.1 | 76.1-86.0 | 91.5 | 89.0-94.1 | 89.9 | 86.7-93.0 |
| Nord Kivu | 97.5 | 96.3-98.7 | 99.3 | 98.7-99.9 | 99.0 | 98.5-99.6 | 99.6 | 99.3-100.0 |
| Province Orientale | 95.2 | 93.2-97.2 | 87.7 | 85.0-90.5 | 95.1 | 93.1-97.1 | 94.9 | 93.5-96.3 |
| Sud Kivu | 94.6 | 92.5-96.6 | 94.9 | 92.8-97.0 | 95.3 | 93.5-97.2 | 96.9 | 95.5-98.2 |

Notes:

a. Data in the table present the adjusted coverage unless stated. Standardized prevalence was calculated after adjusting for women’s age, education attainment, marital status, household heads’ sex, residence region, and household wealth index group.
